# Genotype distribution of methicillin-susceptible *Staphylococcus aureus* clinical isolates in Iran: high multiresistant clonal complex 8

**DOI:** 10.1186/s13104-020-05127-w

**Published:** 2020-06-08

**Authors:** Zahra Tayebi, Hossein Goudarzi, Masoud Dadashi, Mehdi Goudarzi

**Affiliations:** 1 Microbiology Department, Tehran Medical Sciences Branch, Islamic Azad University, Tehran, Iran; 2 Department of Microbiology, School of Medicine, Shahid Beheshti University of Medical Sciences, Tehran, Iran; 3 Department of Microbiology, School of Medicine, Alborz University of Medical Sciences, Karaj, Iran

**Keywords:** *Staphylococcus aureus*, Methicillin-susceptible *S. aureus*, *agr* allotype, *spa*

## Abstract

**Objective:**

Compared to methicillin-resistant *Staphylococcus aureus* (MRSA), there have been few studies focused on the molecular characterization of methicillin-susceptible *Staphylococcus aureus* (MSSA). In this cross-sectional study, 85 MSSA isolates were characterized by antimicrobial susceptibility testing, virulence genes analysis, accessory gene regulator (*agr*) typing, and *S. aureus* protein A locus (*spa*) typing.

**Results:**

In present study, 9 different clonal complexes namely CC8-MSSA-t037 (22.4%), CC8-MSSA-t008 (11.8%), CC7-MSSA-t091 and CC30-MSSA-t021 (each 9.4%), CC8-MSSA-t037 (8.3%), CC398-MSSA-t034 (7.1%), CC22-MSSA-t005 (5.9%), CC5-MSSA-t002 and CC15-MSSA-t084 (each 4.7%), CC22-MSSA-t790 and CC59-MSSA-t437 (each 3.5%), CC22-MSSA-t1869, CC5-MSSA-t045, and CC45-MSSA-t015 (each 2.3%), CC30-MSSA-t318 and CC15-MSSA-t491 (each 1.2%) were found. *agr* types detected in tested strains were mainly type I (76.5%), II (12.9%), and III (10.6%). Of 85 MSSA examined isolates, 48 (56.5%) isolates were toxinogenic with 27 producing *pvl* (31.8%) and 21 *tst* (24.7%). The findings of the study show a high genetic diversity in MSSA strains warranting continued surveillance to provide critical insights into control and treatment of MSSA infections.

## Introduction

*Staphylococcus aureus* is a common hospital- and community-acquired pathogen [1]. It is responsible for a multitude of human infections ranging from minor skin and soft tissue infections to serious and life-threatening conditions [2]. Although the epidemiology of *S. aureus* strain diversity appears to differ by geographic region, there has been a dramatic increase in the prevalence of *S. aureus* strains associated with human infections around the world and this appears to be especially true for methicillin-susceptible Staphylococcus aureus (MSSA) [3–5]. MSSA is a challenge for health-care settings and is becoming a public health concern [1, 6]. Compelling evidence suggests virulence genes may play an important role in serious MSSA infections, which are further exacerbated by the widespread circulation and emergence of drug-resistant strains [3]. Antimicrobial resistance is a barrier to successful control of *S. aureus* infections [7, 8]. Knowledge of genetic variability, clonal relatedness, and dissemination of staphylococcal infections may help to provide crucial insight for implementation of infection control programs, rational use of antibiotics, and better understand the evolution of these species [9, 10]. Although there is information about characteristics of MSSA strains in Iran, limited attention has been given to clonal diversity, and virulence gene prevalence among strains. To address these data limitations, the current study was performed to investigate the genetic background of MSSA strains isolated from patients.

## Main text

### Methods

#### Bacterial isolates

Eighty-five MSSA isolates were obtained from hospitalized patients at four hospitals affiliated to Shahid Beheshti University of Medical Sciences during an 9-month collection period from March 2019 to November 2019. This study protocol was approved by the Ethics Committee of the Shahid Beheshti University of Medical Sciences in Tehran, Iran (IR. SBMU. MSP.REC. 1398. 774). Furthermore, we confirmed *S. aureus* isolates phenotypically by using standard microbiological techniques. Polymerase chain reaction (PCR) assay targeting the *S. aureus*-specific *nuc* gene was applied to verify the isolates [11, 12]. The *S. aureus* isolates susceptible to cefoxitin disc (30 µg, Mast Co., UK) in a disc diffusion assay using established methods (CLSI 2018) and negative for the presence of *mecA* gene by PCR were considered as MSSA strains [12].

#### Evaluation of antimicrobial activities

In present study, Kirby–Bauer disk diffusion method was applied to determine the antimicrobial susceptibility of isolates based on the clinical and laboratory standards institute (CLSI) criteria (CLSI 2018). The antimicrobial agents included penicillin, teicoplanin, gentamicin, kanamycin, amikacin, tobramycin, clindamycin, erythromycin, tetracycline, linezolid, rifampicin, mupirocin, ciprofloxacin, quinupristin–dalfopristin, and trimethoprim–sulfamethoxazole (Mast Co., UK). The minimal inhibitory concentrations (MIC) values of vancomycin was evaluated by broth microdilution method. Susceptibility test was quality controlled by using *S. aureus* ATCC 25923, ATCC 43300, and ATCC 29213 strains.

#### DNA isolation and screening of the key virulence related genes

Genomic DNA was isolated using the phenol–chloroform extraction method. All of the isolates were screened for virulence encoding genes namely: exfoliative toxin genes (*eta*, and *etb*), Panton-Valentine leukotoxin gene (*pvl*), and toxic shock syndrome toxin (*tsst*-*1*) by PCR assay [12–14]. The *S. aureus* ATCC49775 and toxin positive *S. aureus* strains obtained from our previous were used as reference strains [14]. The *S. aureus* strain ATCC 25923 were also used as negative control.

#### Molecular typing methods

Multiplex PCR was performed for *agr* type detection using primer set comprising a common forward primer (Pan) and reverse primers (*agr*1, *agr*2, *agr*3, and *agr*4) specific to each *agr* group [15]. *agr* types were identified by comparing the banding patterns of isolates to RN6390 (*agr* type I), RN6607 (*agr* type II), RN8465 (*agr* type III), RN4550 (*agr* type IV), and RN6911 (negative control), as reference strains. PCR amplification was used for *spa* typing as described previously [16]. In this method polymorphic X region of *spa* gene amplified by PCR with forward (5′-AGACGATCCTTCGGTGAGC-3′) and reverse (5′-GCTTTTGCAATGTCATTTACTG-3′) primers. The purified PCR products were sequenced and then edited. The Ridom SpaServer database (http://www.spaserver.ridom.de) was applied to determine the *spa* type of each isolate. Each set of PCR reactions include a *spa*-type t008 isolate from our previous study as positive control sample (14), and a reaction mixture containing no template DNA as a control for possible false positive results.

### Results

The sources of isolates included: skin and soft tissue wounds (44.7%), purulent exudates from wounds or abscesses (17.7%), urine (14.1%), blood (11.8%), sputum (8.2%), and other body fluids (3.5%). Out of 85 MSSA isolates, 29 isolates were obtained from hospital H1 (34.1%), 25 isolates from hospital H2 (29.4%), 20 isolates from hospital H3 (23.5%), and 11 isolates from hospital H4 (13%). Among 85 MSSA isolates tested, the highest rate of resistance was detected for penicillin (74.1%), and gentamicin (54.1%). (Table 1). All isolates were susceptible to linezolid, teicoplanin, and vancomycin. Totally, 12 resistance patterns were identified. Multidrug resistance (MDR) represented 69.4% of the isolates examined in present research. Inducible and constitutive resistance to clindamycin were detected in 12 (14.1%), and 29 (34.1%) of the isolates tested. Of 85 MSSA examined isolates, 48 (56.5%) were toxinogenic with 27 producing *pvl* (31.8%) and 21 *tst* (24.7%).

**Table 1:** Resistant pattern and distribution of samples in 85 MSSA strains isolated from clinical sources

Number of antibiotic classes	Antibiotic resistance pattern, no (%)	Number of isolates (%)	Samples (no, %)
7	PEN, GEN, KAN, AMK, ERY, CLI, TET	15	W (5, 33.4), P (6, 40), S (2, 13.3), B (2, 13.3)
	PEN, KAN, CLI, ERY, CIP, SYN, MUP	6	W (6, 100)
6	PEN, GEN, AMK, CLI, ERY, CIP	8	W (2, 25), P (6, 75)
5	AMK, ERY, TET, CIP, RIF	10	W (4, 40), P (2, 20), B (2, 20), S (2, 20)
	PEN, GEN, TOB, SXT, SYN	3	U (3, 100)
	PEN, KAN, ERY, TET, MUP	2	W (2, 100)
4	PEN, GEN, TOB, TET	9	W (4, 44.5), S (2, 22.2), B (3, 33.3)
	PEN, GEN, CLI, SYN	1	B (1, 100)
3	PEN, AMK, RIF	5	W (5, 100)
2	PEN, GEN	10	W (4, 40), B (2, 20), P (1, 10), BF (3, 30)
	CIP, SXT	9	U (9, 100)
1	PEN	4	W (4, 100)
Without resistance	–	3	W (2, 66.7), S (1, 33.3)

*PEN* penicillin, *ERY* erythromycin, *TET* tetracycline, *CLI* clindamycin, *GEN* gentamicin, *SXT* trimethoprim–sulfamethoxazole, *CIP* ciprofloxacin, *SYN* quinupristin–dalfopristin, *TOB* tobramycin, *AMK* amikacin, *RIF* rifampicin, *KAN* kanamycin, *MUP* mupirocin, *W* wound, *P* pus, *U* urine, *B* blood, *S* sputum, *BF* other body fluids

*agr* typing discriminated the 85 MSSA isolates in 3 *agr* type namely I (76.5%), II (12.9%), and III (10.6%). *spa* results showed 16 types corresponding to nine clonal complexes (CCs), namely CC8 (42.3%), CC22 (11.8%), CC30 (10.6%), CC7 (9.4%), CC5 (7.1%), CC398 (7.1%), CC15 (5.9%), CC59 (3.5%), and CC45 (2.3%). *spa* type t037 was the most common *spa* type identified among 85 MSSA isolates, with a frequency of 22.3%, followed by t008 (11.8%), t021 and t091 (each 9.4%), t030 (8.2%), t034 (7%), t005 (5.9%), t084 and t002 (each 4.7%), t790 and t437 (each 3.5%), t1869, t045, and t015 (each 2.4%), t318 and t491 (each 1.2%) (Table 2). All the mupirocin resistant strains belonged to CC8-MSSA type.

**Table 2:** Molecular characterization of MSSA strains isolated from patients

Clonal complex (CC)	*spa* types (no; %)	*agr* type	Virulence genes (no; %)	Hospitals (no; %)	Total N (%)
CC8	t037 (19; 73.1), t030 (7; 26.9)	I	*tst* (15; 57.7)	H1 (8; 30.8), H2 (6; 23.1), H3 (10; 38.5), H4 (2; 7.6)	26 (30.5)
	t008 (10; 100)	I	*pvl* (10; 100)	H1 (4; 40), H2 (2; 20), H3 (4; 40)	10 (11.8)
CC22	t005 (5; 50), t790 (3; 30), t1869 (2; 20)	I	*pvl* (5; 50), *tst* (3; 30)	H1 (4; 40), H2 (6; 60)	10 (11.8)
CC30	t021 (8; 88.9), t318 (1; 11.1)	III	*pvl* (8; 88.9)	H1 (3; 33.3), H2 (3; 33.3), H3 (2; 22.3), H4 (1; 11.1)	9 (10.6)
CC7	t091 (8; 100)	I	–	H1 (4; 50), H2 (4; 50)	8 (9.4)
CC5	t002 (4; 66.7), t045 (2; 33.3)	II	–	H1 (1; 16.7), H2 (2; 33.3), H3 (1; 16.7), H4 (2; 33.3)	6 (7.1)
CC398	t034 (6; 100)	I	–	H1 (5; 83.3), H3 (1; 16.7)	6 (7.1)
CC15	t084 (4; 80), t491 (1; 20)	II	*pvl* (4; 80)	H4 (5; 100)	5 (5.9)
CC59	t437 (3; 100)	I	*tst* (3; 100)	H2 (2; 66.7), H4 (1; 33.3)	3 (3.5)
CC45	t015 (2; 100)	I	–	H3 (2; 100)	2 (2.3)

Inducible clindamycin resistance was observed in CC8-MSSA-t037 (n = 5), CC30-MSSA-t021 (n = 4) CC8-MSSA-t008 (n = 3) isolates; while constitutive resistance phenotype was observed in CC8-MSSA-t037 (n = 8), CC8-MSSA-t030 (n = 3), CC-MSSA-t034 (n = 4), CC-MSSA-t091 (n = 5), CC-MSSA-t045 (n = 4), CC-MSSA-t005 (n = 4), and CC-MSSA-t790 (n = 1) isolates. Detailed results of the genotype distribution are presented in Table 2.

### Discussion

Infection with MSSA as the most common pathogen in hospitalized patients is becoming increasingly problematic globally and requires special attention [3]. As presented in Table 2, our results indicated 9 different clonal complexes and 16 *spa* types among MSSA isolates. We detected CC8 in 42.3% of isolates. Although multi-resistant CC8 was previously reported as one of the main international CCs of MRSA, the predominance of the CC8-MSSA clone has previously been reported in Europe [17] and Africa [18, 19]. We showed that all the mupirocin resistant strains belonged to CC8-MSSA clone. In this connection, similar findings have been reported from Kuwait [20], Ireland [21], and Nigeria [22]. We confirmed the presence of CC22 as the second dominant MSSA genotypes (11.8%). This clone is widely spread both as MSSA and MRSA in Kuwait, China, Ireland, the United Arab Emirates, Japan, Korea, and Australia [3, 20, 21]. As mentioned, the frequency of CC30 was found to be 10.6%. The presence of ST30-MSSA, known as the Southwest Pacific clone, has been noted in Australia, the UK, Germany, Lebanon, Abu Dhabi, and Kuwait [21, 23].

We noted a relatively low prevalence of CC7 (9.4%), CC5 (7.1%), CC398 (7.1%), CC15 (5.9%), CC59 (3.5%), and CC45 (2.3%) in our study. A study conducted in China during the 4-year period indicated that CC22-t309 (26.0%), CC188-t189 (5.1%), CC796-t796 (4.8%), CC121-t435 (4.8%), and CC398-t571 (3.6%) were the most dominant clones among MSSA isolates [3]. Ahigh diversity of MSSA isolates was reported by Uzunovic et al. from Bosnia and Herzegovina [24]. They demonstrated that MSSA clones were associated with 14 CCs with the majority of CC42, CC22, CC5, and CC30. A 2015 study in Korea showed that the most prevalent MSSA clones were CC72 (29.3%), followed by CC188 (21.9%), CC121 (19.5%) and CC30 (9.6%) [25]. A recent study by Shore et al. (2014) in Ireland also showed that CC22, CC30, and CC121 were found in MSSA isolates [26].

Present results demonstrated that out of the 85 tested MSSA, 27 isolates (31.8%) carried *pvl* encoding gene. Prevalence PVL positive MSSA strains has varied among studies from different geographic regions including China (34.4%) [3], Lebanon (12%) [23], Ireland (17%) [26], Colombia (32.3%) [4], England (60%) [27], Africa (57%) [22], Russia (55%) [6]. In a recent study conducted during a 9-year period in Ireland, Shore et al. indicated a decreasing trend of PVL among MSSA strains (20% to 2.5%) [26]. However, there are reports that indicate greater potential of MSSA strains to secrete toxins, such as PVL implying the important role PVL-MSSA strains generally play as reservoirs for highly virulent PVL-positive MRSA clones [16, 19]. In agreement with other studies [3, 18, 26, 28], high genetic diversity of PVL-positive MSSA isolates belonging to CC8 (11.8%), CC30 (9.4%), CC/22 (5.9%), and CC/15 (4.7%) clones were observed in our study. This finding supports previous result from UK in which CC22, CC88, CC30, and CC1 were detected as major sequence types among PVL-positive MSSA isolates [27]. Shore et al’s study on 39 PVL-positive MSSA isolates in Ireland depicted that CC30 was the dominant clone (38.5%), followed by CC22 (25.6%), CC121 (18%), CC1 (10.3%), and CC88 (7.7%) clones [26]. Another study from Greece reported that PVL-positive MSSA belonged to ST14, ST97 and ST101 [11].

In our strain collection, 24.7% of isolates were *tst* positive. This reported rate was different from the earlier studies in Africa (7%) [19], China (4%) [29] and Turkey (14.2%) [30]. Contrary to earlier studies [11, 31] which indicated CC30 MSSA as a prevalent lineage, our MSSA isolates harboring *tst* gene were associated with CC8-MSSA-t037 (11.8%), CC8-MSSA-t0307 (5.9%), CC22-MSSA-t790 (3.5%), and CC59-MSSA-t437 (3.5%) clones. A multicenter study from china reported that 4.0% of MSSA isolates carried *tst* gene which belonged to CC5 clone [29]. A recent study from Greece, displayed that *tst*-positive MSSA was distributed into four STs and the majority of them belonged to CC30 clone [11]. A recent systematic review and meta-analysis study in Iran reported a relatively high prevalence of the *tst* encoding gene among *S. aureus* clinical isolates (21.3%) [32]. In a study conducted by Motamedifar et al. (2015), the carriage rates of *tst* were significantly higher in MSSA isolates in comparison to MRSA isolates (18.1% vs. 11.6%) [33]. Overall, distribution of *tst*-positive MSSA clones seems geographically different and CC8 may represent a newly emerging clone in Iran.

According to the evidence, the *agr* genotypes are strictly associated with the clonal lineages [34, 35]. In the current study, the *agr* type I, as the most predominant type (76.4%), was associated with CC8, CC22, CC7, CC45, CC398, and CC59 isolates. In line with our results, Croes et al. demonstrated that CC7, CC8, CC22, CC25 and CC45 clonal lineages harbored *agr* I [7]. A study conducted by Zhao et al. in China demonstrated a high prevalence of *agr* type I (68.8%), followed by *agr* III (18.7%), and *agr* IV (12.5%) among MSSA isolates [9].

## Limitations

Our research had some limitations. Firstly, present work lacks detailed clinical information about the patients, Secondly, our samples were not collected consecutively. Thirdly, whole genome sequencing technique was not applied in the present work due to some of technical limitations.

## Data Availability

The datasets generated and analyzed during this study were included in the main document of this manuscript.

## Abbreviations

PVL: Panton-Valentine leukotoxin
TST: Toxic shock syndrome toxin
MDR: Multidrug resistance
*spa*: Surface protein A
MLST: Multi-locus sequence typing
*agr*: Accessory gene regulator
PCR: Polymerase chain reaction
MRSA: Methicillin-resistant *S. aureus*
MSSA: Methicillin-sensitive *S. aureus*
